# Multiple Biological Roles of Extracellular Vesicles in Lung Injury and Inflammation Microenvironment

**DOI:** 10.1155/2020/5608382

**Published:** 2020-07-14

**Authors:** Gang Su, Xiaohong Ma, Haidong Wei

**Affiliations:** ^1^Institute of Genetics, School of Basic Medical Sciences, Lanzhou University, Lanzhou, China; ^2^Lanzhou University Second Hospital, Lanzhou University, Lanzhou, China

## Abstract

Lung injury and inflammation are complex pathological processes. The influence and crosstalk between various cells form a characteristic microenvironment. Extracellular vesicles from different cell sources in the microenvironment carry multiple cargo molecules, which affect the pathological process through different pathways. Here, we mainly discussed the mechanism of crosstalk between alveolar epithelial cells and different immune cells through extracellular vesicles in lung inflammation and reviewed the mechanism of extracellular vesicles released by blood and airways on lung inflammation. Finally, the role of extracellular vesicles in viral infection of the lung was also described.

## 1. Introduction

The lung is an important part of the respiratory tract and the organism, which is susceptible to inflammation and damage caused by microbes or systemic diseases. It is not fully understood how the inflammation and injury of the lungs, especially those caused by noninfectious stimuli, are initiated and transmitted. Crosstalk between infected and stressed lung epithelial cells and immune cells forms a characteristic microenvironment. Stress signals are transmitted between microenvironment cells and the system level, and they are involved in the inflammatory process [1].

Extracellular vesicles (EVs) are actually the key messengers of intercellular communication in these microenvironments [2]. EVs contain different cargoes (such as proteins, RNA, DNA, and lipids) for intercellular transmission at the paracrine and systemic levels. EVs can be released due to cell activation, hypoxia, radiation, damage, complement protein exposure, and cellular stress [3, 4]. According to different biological mechanisms, EVs include exosomes, shed microvesicles (sMVS), and apoptotic bodies [5]. However, the subtypes of different EVs cannot be completely separated according to size or density, because of the overlapping physical characteristics [6]. Exosomes originate from the process of endocytosis and are formed by releasing multivesicular bodies (MVBs) into the extracellular environment [7]. sMVs are formed by the rearrangement of the actin cytoskeleton and buds directly from the plasma membrane [8]. Apoptotic bodies are vesicular bodies formed by atrophy and fragmentation of cells during apoptosis [9]. Although exosomes and sMVs have different biogenesis and membrane origin, their functions appear to be similar after they are released outside the cell [10].

Many lung cell types, including epithelial cells and endothelial cells, as well as infiltrating macrophages, can produce EVs. In most cases, these EVs promote the inflammatory process. However, the functions of extracellular vesicles are complex and dynamic in the process of inflammation. This review focuses on recent studies that have explored the role of EVs in crosstalk between microenvironmental and systemic levels during lung inflammation and injury.

## 2. Effects of Epithelial Cells Releasing EVs on the Inflammatory Process in the Lung Microenvironment

Under the action of stimulating factors, respiratory epithelial cells release extracellular vesicles with pathological characteristics and participate in signal pathways of target cells to stimulate the inflammatory response [11]. For example, mouse lung epithelial cells release exosomes carrying annexin A2, activate through the NF-*κ*B pathway and produce proinflammatory cytokine IL-6, and participate in pulmonary fibrosis and inflammation caused by the anticancer drug Bleomycin reaction [12]. Experiments using A549 cell transfection and phagocytosis confirmed that IFN-*γ* induced p11 expression can promote the release of exosomes carrying ANXA2. This process upregulated the expression of ANXA2 on the surface of lung epithelial cells, enhancing its phagocytosis of apoptotic cells [13].

The process of lung inflammation is the result of intercellular crosstalk in the microenvironment. Extracellular vesicles as an information carrier are involved in the cellular communication between pulmonary epithelial cells and alveolar macrophages and form an important part of lung injury. Diffuse alveolar damage (DAD) and lung epithelial cell death associated with pulmonary inflammation could prompt lung epithelial cells to release vesicles to nearby or distant macrophages, thereby triggering or transmitting inflammatory responses. After hyperoxic stress, lung epithelial cells produced a large amount of EV through ER stress. These EVs encapsulate caspase-3, activate alveolar macrophages through the ROCK1 pathway, and increase their secretion of proinflammatory cytokines and macrophage inflammatory protein 2 (MIP-2) to be involved in lung injury [14]. Combined with in vitro and in vivo experimental methods, it was confirmed that IL-13 can promote the release of epithelial cell-derived exosomes and induces proliferation and chemotaxis of undifferentiated macrophages in the lung during asthmatic inflammation [15]. LPS stimulation can induce respiratory epithelial cells to release exosomes containing prolyl endopeptidase (PE), which is an important regulator of lung remodeling and airway inflammation. Transfected cells with siRNA and knockout mice confirmed that this pathway is achieved through the activation of Toll-like receptor 4(TLR4) [16].

In addition to carrying protein peptides, EVs also contain nucleic acids, such as microRNA, and they are associated with lung injury and inflammation [17]. Hyperoxia stress upregulates the expression of certain miRNAs in epithelial EVs, such as miR-320a and miR-221. These EVs promote macrophage activation, which mediates the inflammatory response in the lungs [18]. Experiments confirmed that EVs isolated from bronchoalveolar lavage fluid (BALF) were derived from alveolar epithelial type I cells (ATIs). These miRNA-containing ATI-EVs are delivered to alveolar macrophages, activate inflammatory factors to polarize the macrophages to M1, and participate in pneumonia caused by Pseudomonas aeruginosa. It is worth noting that these miRNA-rich EVs contained Caveolin-1, a lipid raft protein that may be a biomarker of EV-miRNA enrichment [19]. In addition to miRNA, it has been shown that mitochondrial DNA fragments could be involved in the inflammation through EVs. Low levels of oxidative stress caused by cigarette smoke exposure can damage mtDNA in lung epithelial cells. mtDNA fragments enter naive (nonoxidative stress) epithelial cells through exosomes, activating the ZBP1(Z-DNA binding protein 1)/TBKI(TANK-binding kinase 1)/IRF3(interferon regulatory factor 3) pathway to induce inflammation [20].

## 3. Two-Way Regulation of Immune Cell-Derived Exosomes on Inflammatory Process in the Lung Microenvironment

The interaction of immune cells with alveolar epithelium maintains the alveolar homeostasis. They communicate with each other through cell surface receptors, gap junction channels, release, uptake of secreted EVs, and cytokine signaling [21]. EVs crosstalk between innate immune cells and structural cells to promote inflammation. In chronic inflammation, activated neutrophils release CD63+/CD66b+ exosomes. These exosomes degrade the extracellular matrix (ECM) through integrin Mac-1 and neutrophil elastase (NE), causing chronic obstructive pulmonary disease (COPD) [22]. The hemorrhagic shock (HS)mouse model and the cell hypoxia-reoxygenation model confirmed that exosomes released from HS-activated alveolar macrophage (AM*ϕ*) induce NADPH oxidase-derived reactive oxygen species (ROS) production inside polymorphonuclear neutrophils (PMNs) and subsequent promotion of necroptosis. This experiment revealed the mechanism of pulmonary inflammation caused by HS [23].

EVs released by immune cells can also affect the inflammatory microenvironment through the included miRNAs. After airway inflammation, infiltrating immune cells released miR-223 and miR-142a-containing extracellular RNA (ex-miRNA) and EVs in the inflamed tissue to change the local microenvironment [24]. A lipopolysaccharide (LPS) induced mouse model of acute septic lung injury confirmed that exosomes released by bronchoalveolar lavage fluid (BAL) in the lungs pack miRNAs and cytokines involved in regulating inflammation. Using a coculture model, these exosomes derived from macrophages disrupt the structural barrier by influencing the expression of tight junction proteins in bronchial epithelial cells and promote the inflammatory response [25].

It should be noted that the maintenance of alveolar microenvironment homeostasis also depends on the two-way regulation of immune cells. Some experiments have confirmed that alveolar macrophages secrete exosomes and particles that encapsulate SOCS1 and 3. The SOCS protein family is the endogenous braking factor of JAK-STAT signaling pathway. Alveolar epithelial cells can inhibit the activation of STAT after taking up the exosomes. This SOCS1 and -3 released into the extracellular space through vesicles, by inhibiting intracellular STAT1 and STAT3 signaling, to mediate the crosstalk between macrophages and epithelial cells, is a new way to control inflammation and immune response [26]. Lung epithelial cell (LEPC)-derived IL-25 can affect the expression of Rab27a and Rab27b in lung macrophages (AM*ϕ*), negatively regulate LPS-induced exosomes released by AM*ϕ*, and attenuate the expression of TNF-*α* induced by exosomes [27]. It can be seen that the signal transmission of lung epithelial cells and macrophages through extracellular vesicles maintains a dynamic balance between microenvironments. However, this state may be disturbed under stimulation such as smoking to advance the inflammatory state.

## 4. Circulating Exosomes Participate in Inflammatory Damage in the Microenvironment

In addition to intercellular communication in the local microenvironment, pulmonary inflammatory lesions are also a local response to stress conditions in the body. Exosomes released by the circulatory system activated immune cells through their cargo molecules to participate in the inflammatory response. Such as high oxygen stress will further promote the increase of serum circulating EVs and activate systemic macrophages outside the alveoli, eventually mediating the inflammatory response and causing lung injury [3]. Exosomes from sera of sepsis mice induce cytokines to induce Th1 and Th2 cell differentiation and enhance T lymphocyte proliferation and migration [28]. After acute lung injury (ALI), serum-derived exosomes transfer miR-155 to macrophages, activate nuclear factor *κ*B (NF-*κ*B), and produce tumor necrosis factor *α* (TNF-*α*) and interleukin-6, which promotes macrophage proliferation and inflammatory response [29]. miRNA-126 carried by serum exosomes in patients with allergic asthma is involved in the infiltration of inflammatory cells [30]. Taurocholic acid-induced lung injury in rats with acute pancreatitis showed that circulating exosomes after stress can effectively reach the alveolar compartment and activate lung macrophages as a proinflammatory phenotype [31]. Cigarette smoke (CS) exposure can release circulating exosomes with specific miRNAs to affect the clearance of apoptotic cells by macrophages, participate in endothelial damage and inflammation-related diseases in smokers [32]. As an important part of the systemic circulatory system, lymphoid-derived exosomes are also involved in pulmonary inflammation. Inflammatory disorders following hemorrhagic shock (HS) can cause acute lung injury (ALI). The mechanism is driven by intestinal-derived inflammatory mediators after HS, which promotes exosomes derived from the supernatant of mesenteric lymph node extracts and triggers activation of alveolar macrophages by Toll-like receptor 4 to produce proinflammatory cytokines [33].

The release of circulating exosomes is very important for the inflammatory response. Studies have shown that Rab27a and Rab27b double knockout (Rab27DKO) mice have insufficient secretion of circulating exosomes, have a chronic inflammatory phenotype, and have increased inflammatory cytokines and bone marrow proliferation. Rab27-dependent release of circulating exosomes contributes to homeostasis within the hematopoietic system and appropriate responsiveness to inflammatory stimuli [34]. Overexpression of serum exosome miR-103-3p can attenuate the inflammatory response induced by lipopolysaccharide. Using luciferase reporter assay and immunoprecipitation technology, it was confirmed that miR-103a-3p directly binds to transducin *β*-like 1X-related protein 1 (TBL1XR1), mediating the NF-*κ*B signaling pathway, thereby regulating the immune response [35].

In addition, circulating exosomes in the respiratory tract are also involved in the inflammatory process. The exosomes isolated from inflammation-mediated bronchoalveolar lavage fluid (BALF) are complex and contain many cargo molecules related to inflammation. Proteomics comparison of BALF-derived exosomes from cystic fibrosis (CF) and asthma patients found higher levels of antioxidant proteins (superoxide dismutase, peroxidase, etc.) and those involved in leukocyte chemotaxis. Exosomes regulate the inflammatory process by carrying proteins, preventing excessive inflammation [36]. BALF-derived exosomes from asthma patients promote inflammation by increasing the production of cytokines and leukotriene-4 in airway epithelium [37]. After exposure to house-dust mite (HDM), using miRNA microarrays confirmed that airway-secreted EVs (AEVs) isolated from BALF contained increased Th2 inhibitory miRNAs, which promoted airway secretion and be involved in the allergic airway inflammation [38]. This miRNA secreted into mouse BALF microvesicles has significantly upregulated miR-223 and miR-142, activates lung macrophages through Nlrp3 inflammatory bodies, and mediates pulmonary inflammation [39]. Analysis of tracheal aspiration fluids (TAs) in preterm infants with oxygenation and mechanical ventilation showed that this AEVs originated from mast cells. AEVs can mediate the release of *β*-hexosaminidase (*β*-hex) associated with inflammation and participate in the inflammation of the lungs of chronic preterm infants caused by oxygen poisoning [40]. In a mouse model of congenital inflammation lacking the Toll-like receptor TLR3-NF*κ*B/RelA, BALF-derived exosomes are rich in coagulation factors and are involved in inflammation through interstitial fibrin deposition [41]. In addition, changes in the lipid composition of AEVs, such as higher levels of sphingomyelin in exosomes derived from asthma patients, significantly reduced phosphatidylglycerol, ceramide-phosphate, and ceramide, are associated with chronic airway inflammation [42].

## 5. Extracellular Vesicles Are Messengers and Vectors of Virally Infected Lung Inflammation

Airway and alveolar epithelial cells are targets for viral infections including rhinovirus, influenza virus, coronavirus, and pneumonia virus. Infected epithelial cells issue an innate cellular antiviral response, coordinating myeloid and lymphoid cells to participate in the immune process, which can be achieved by the EVs pathway. For example, human rhinovirus (RV) infects bronchial epithelial cells, activates Toll-like receptor (TLR)3, produces immunoregulatory extracellular matrix protein proinflammatory tenascin-C and releases small extracellular vesicles (sEV). These sEVs may enhance airway inflammation and regulate immune response to infection [43]. Exosomes isolated from respiratory syncytial virus (RSV)-infected cells can activate the innate immune response by inducing human monocytes and airway epithelial cells to release cytokines and chemokines [21].

Extracellular vesicles are not only involved in the inflammatory process following a viral response but are also a powerful vector for transmitting viral particles and viral genomes in bulk between organisms [44]. Vesicles transport large numbers of mature infectious virus particles between cells, which can increase the infectivity to host cells [45]. Cluster packaging of vesicles by multiple single-stranded RNA virus particles, including rhinoviruses, allows multiple viral RNA genomes to be collectively transferred to a single cell, promoting genetic replication between viruses and quasispecies [46]. This observation offers a new insight into the mechanism of virus transmission.

## 6. Conclusions

**Figure 1:**
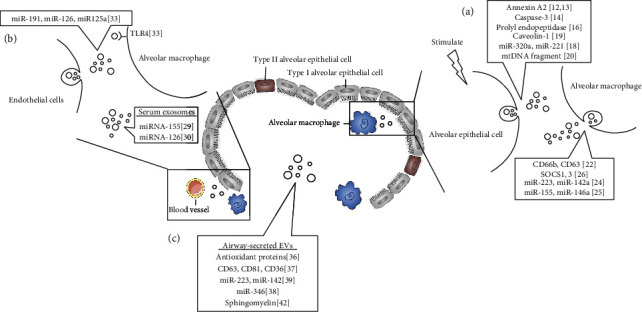
Information transfer and cargo carried by extracellular vesicles in the inflammatory microenvironment of the lungs. (a) Communication and cargo molecules of extracellular vesicles derived from alveolar epithelial cells and alveolar macrophages. (b) Information transmission of circulating extracellular vesicles. (c) The respiratory tract secretes cargo molecules of extracellular vesicles.

Extracellular vesicles of different origins provide multiple biological roles in complex lung inflammatory injury. Cell-derived EVs of the lung microenvironment and the circulatory system disrupt the original microecological balance and promote the inflammatory process by transmitting inflammation-related proteins and nucleic acids (Figure 1). An in-depth understanding of the structural characteristics and biological functions of EVs is of great significance for elucidating the pathogenesis of pulmonary inflammation, the transmission of infection, and providing new treatment strategies.
